# Evaluating the Role of Maternal and Paternal Trauma Exposure, Emotional Security, and Mental Health in Predicting Psychological Adjustment among Palestinian Adolescents

**DOI:** 10.3390/ijerph19159288

**Published:** 2022-07-29

**Authors:** Catherine A. Maloney, Laura E. Miller-Graff, Bethany Wentz, Edward Mark Cummings

**Affiliations:** 1 Department of Clinical Psychology, University of Notre Dame, Notre Dame, IN 46556, USA; lmiller8@nd.edu (L.E.M.-G.); bwentz@nd.edu (B.W.); ecumming@nd.edu (E.M.C.); 2 Kroc Institute for International Peace Studies, University of Notre Dame, Notre Dame, IN 46556, USA

**Keywords:** violence, mental health, psychological adjustment, emotional security, Palestine

## Abstract

Previous research has established a strong relationship between family system functioning and child adjustment outcomes. However, within the context of Gaza, an evaluation of both maternal and paternal factors associated with adolescent psychological adjustment has not yet been thoroughly evaluated. The current study examines how maternal and paternal trauma exposure, emotional security, and mental health are related to parent-reported scores of adolescent psychological adjustment, while controlling for adolescent trauma exposure and security in the family. The sample included *N* = 68 family units living in the Gaza Strip, with two parents and one adolescent surveyed within each unit (adolescent *M_age_* = 14.03 years). The regression model examining maternal factors was significant overall (*F* = 7.44, *R*^2^ = 42.70%, *p* < 0.001), with increased maternal depression associated with greater adolescent adjustment difficulties (*β* = 0.31, *p* = 0.011) and greater maternal emotional security in the family associated with fewer adolescent adjustment difficulties (*β* = −0.36, *p* = 0.004). The regression model examining paternal factors was also significant (*F* = 4.57, *R*^2^ = 31.00%, *p* < 0.001), with increased paternal trauma exposure associated with greater adolescent adjustment difficulties (*β* = 0.32, *p* = 0.012). Understanding family-level factors associated with adolescent adjustment is an important step in conceptualizing the mental health needs of conflict-affected youth within the context of Gaza and more broadly.

## 1. Introduction

Worldwide, approximately 452 million children live in an area affected by armed conflict [1]. Of these children, about 1 million live in the Gaza strip and consistently report high rates of exposure to violence [2]. For example, a study of Gazan youth found that 94.9% had directly experienced tear gassing, 97.0% witnessed a shooting, and 51.7% had witnessed someone being killed or seriously injured [3]. Another study of Gazan adolescents found that 98% of the sample reported exposure to five or more traumatic events [4]. Previous research has documented well-known adverse effects of such violence on Palestinian youth across Gaza and the West Bank, including the development of posttraumatic stress, somatic symptoms, anxiety, depression, aggression, and other behavioral and emotional concerns [5,6,7,8,9,10,11]. Studies have also begun to examine mediators and moderators of the relation between youth trauma and adjustment in Palestine, including factors such as identity, self-esteem, positive parenting, and family support [9]. Much of the available research, however, includes data integrated from a single reporting source (i.e., maternal reports), while little research includes the perspective of both parents, including fathers. This gap limits our understanding of family wide relations between parent experiences, family processes, and adolescent psychological adjustment. The objective of the current study was to examine the associations between Gazan mothers’ and fathers’ traumatic experiences, mental health, and family-wide emotional security with adolescent psychological adjustment, in order to more thoroughly understand how intergenerational experiences may influence adolescent adjustment outcomes. The context of Palestine was selected for the current study because the area has been characterized by chronic and extended sociopolitical violence. Additionally, the authors of the current study have established strong community collaborations and partnership, which are essential for effective research in such contexts.

In examining associations of parental trauma exposure, mental health, and emotional security with adolescent adjustment, it is important to contextualize the experiences of families living in Gaza. In addition to facing direct violence, generations of families in Gaza have faced numerous forms of structural violence. Originally coined by Johan Galtung, the term *structural violence* refers to “violence that is ‘built into’ the structure and shows up as unequal power and consequently as unequal life chances” (p. 171) [12,13]. The devastating effects of structural violence are evident across Gaza. The Gaza Strip is about 140 mi^2^ and home to approximately 2 million people. The strip of land is surrounded on two sides by the State of Israel, on one side by Egypt, and on the final side by the Mediterranean Sea. While Egyptian authorities regulate movement across Egypt’s shared border, the Israeli military closely regulates all other Gazan borders, including access to the Sea [14,15]. Gazans must complete an application process with the Israeli government to request passage through checkpoints and must be granted a permit in order to cross the border [14]. The sea is regulated by the military, with designated areas where Palestinian fishermen are permitted to travel and other areas that are restricted [14]. In addition to controlling the movement of the Gazan people, the Israeli government also controls the movement of material goods into Gaza, and the region is chronically impacted by food scarcity, limited electricity, underdeveloped infrastructure, and overcrowding [14,15,16].

In 2007—prior to the living memory of adolescents living in Gaza today—the Israeli government incited a blockade against Gaza in response to Hamas gaining political control of the region [17]. This blockade occurred following an escalation of violence between Hamas and Israel and has resulted in deteriorating infrastructure and devastating humanitarian concerns across Gaza [17]. Since 2007, there have been numerous escalations in direct violence affecting Gazan communities [18]. From July August 2014, violence between Gaza and Israel resulted in the death of 2251 Palestinians, including 551 children, and the destruction of schools, health centers, and houses [18,19]. From 2018–2019, a series of Palestinian protests at the Gaza-Israel border turned violent and led to the death of 214 Palestinians, while an additional 36,000 were injured [20,21]. One of the most recent major escalations, in May 2021, was spurred by Israel’s proposed eviction of Palestinian residents from the Sheikh Jarrah neighborhood in East Jerusalem [18]. The 2021 events led to Israel bombing Gazan homes and schools, and the death of 256 Palestinians, including 66 children [22,23]. Although these events highlight only a few of the larger-scale incursions over the past decade, Gazan adolescents are consistently exposed to examples of direct and structural violence that shape their daily experiences and wellbeing.

### 1.1. Social Ecological and Emotional Security Frameworks

The pathways connecting sociopolitical violence exposure and subsequent adolescent adjustment outcomes (i.e., an individual’s ability to positively adapt to changes within themselves or their environment in a manner that supports their psychological wellbeing) have been largely explored in previous literature through social–ecological and emotional security theories. The social–ecological framework theorizes that multiple levels of a system (i.e., individual, family, community, society) dynamically interact to influence mental health outcomes (e.g., adolescent adjustment) [24,25]. Cummings et al. (2014) highlights that due to the broad nature of the social–ecological model, it is important for researchers to continue to explore narrower elements of the model that are pertinent to the local context and may contribute to outcomes of interest [24]. For example, Dubow et al. (2009) hypothesizes a mediating role of cognitive processes and self-schema in the relationship between ethnopolitical violence and youth adjustment outcomes [25]. Additionally, several papers by Cummings and colleagues (2011; 2015; 2017) consider the mediating role of child emotional security in the relationship between ethnopolitical violence and subsequent adjustment [26,27,28].

Emotional security theory first emerged as a model of the relationship between parents and children, and has since expanded across literature to incorporate broader family and community dynamics that influence child emotional development [26,29]. Cummings et al. (2011) defines emotional security theory as follows:

“…children’s protection, safety and security are core concerns in their regulatory functioning. Accordingly, emotional security is defined as a goal around which functioning is regulated, with emotional (e.g., fear, anger), behavioral (e.g., mediation, withdrawal, intervention) and cognitive (e.g., threat perceptions) responses activated in the service of regaining or maintaining a desired level of emotional security in the face of threats to that goal” (p. 216) [26].

Cummings et al. (2011) discusses the scarcity of research exploring mechanisms through which sociopolitical violence may influence child adjustment, a research gap that has begun to be addressed through the team’s longitudinal study in Northern Ireland, the results of which supports the role of emotional security in mediating the relationship between violence exposure and subsequent adjustment outcomes [26]. Children and adolescents with lower levels of emotional security have been found to exhibit potentially maladaptive responses to external stimuli, such as aggression, which may play a role in the development of poorer adjustment outcomes [26,29]. Therefore, evaluating the relationship between sociopolitical violence exposure, emotional security, and adjustment within local contexts is essential in understanding youth’s psychosocial development.

Beyond emotional security, is also important to consider historical and intergenerational trauma as a potential mechanism within the social–ecological framework of youth adjustment in the Gazan context. Since the current paper examines the impact of mothers’ and fathers’ violence exposure, mental health, and emotional security on their children’s adjustment, it is essential to understand how the impact of collective, historical traumas can transcend generations, even when children did not directly experience traumatic events. The concept of *historical trauma* first began to appear in psychological literature following the atrocities of World War II and originally centered on the experiences of Holocaust survivors and their families [30,31,32]. Since that time, the impact of historical trauma has been largely recognized across groups of people who have faced chronic and systematic oppression and elimination of their communities and culture, such as through colonization, forced acculturation, or genocide [31,33]. Previous research suggests that intergenerational trauma can be transmitted across generations through ongoing systematic inequities that persist across generations as well as through the impact of parental trauma on parent-child relational processes [34]. Ample research exists on the experiences of violence among Palestinian adolescents living in Gaza and the West Bank, as well as the relationship between current violence exposure and mental health outcomes, but Barron and colleagues (2015; 2016) call for additional research on the relationship between intergenerational trauma and young people’s mental health [34,35].

The current paper contributes to our understanding of the social–ecological model of mental health within the context of Gaza, by specifically exploring the *intergenerational* implications of parental trauma exposure (including exposure to ethnopolitical violence), parental emotional security, and parental mental health on adolescent psychological adjustment outcomes. While the link between youth violence exposure and youth mental health difficulties in Palestinian adolescents has been well established, the collective trauma experienced by Palestinians across generations presents a need to understand how mothers’ and fathers’ experiences may independently influence their adolescents’ outcomes within the social–ecological context.

### 1.2. Parental Trauma Exposure

Previous literature has established that adults and youth affected by armed conflict and sociopolitical violence across diverse settings are at heightened risk of developing mental illness and psychosocial difficulties [36,37,38,39]. For young Palestinians, studies across Gaza, the West Bank, and East Jerusalem illustrate that exposure to traumatic events is associated with increased posttraumatic stress symptoms, depression, anxiety, and additional social and emotional problems [8,9,40,41]. This relationship between trauma exposure and mental health and psychosocial difficulties holds true among Palestinian adults as well [42,43,44]. While it has been established that violence exposure affects both adults and children living in the West Bank and Gaza, fewer studies have explored how parental trauma exposure may independently predict child outcomes. However, with a history of chronic and collective violence against Palestinians, it is important to consider how intergenerational trauma informs young Palestinians’ development [34].

Qouta et al. (2005) sampled mothers and children (aged 6–16 years) in Gaza and found that female children were at an increased risk of developing post-traumatic stress symptoms and internalizing concerns when their mothers experienced trauma exposure, while male children were at an increased risk of developing posttraumatic stress symptoms and internalizing concerns when both themselves and their mothers had experienced trauma exposure [45]. However, a study by Palosaari et al. (2013) potentially contradicts this finding, showing that Gazan mothers’ exposure to trauma prior to their children’s birth is associated with fewer depressive symptoms among their children, and was mediated by lower rates of psychological maltreatment (children were 10–12 years of age at the time of the study) [46]. However, the study also shows that fathers’ exposure to trauma prior to their children’s birth is associated with greater depressive and conduct symptoms among their children, as mediated by higher rates of psychological maltreatment [46]. Due to the established impact of sociopolitical violence on youth mental health, and the importance of understanding intergenerational effects of trauma, it is necessary for future research to explore the relationship between parental trauma and youth psychosocial outcomes. This is particularly important in areas such as Gaza, where chronic violence has been ongoing for generations.

### 1.3. Parental Emotional Security

Emotional security has been studied among youth in conflict areas across a variety of settings, and previous research shows that increased emotional security is linked to positive mental health and psychosocial outcomes [26,47]. However, a majority of existing literature focuses primarily on measuring youth’s emotional security within family and community settings, rather than considering how parents’ sense of emotional security may influence their children’s outcomes. The importance of exploring parents’ emotional security is evident based on previous research. For example, Punamäki et al. (2017), which shows that Gazan families categorized as experiencing “insecurity and problematic relationships” were more likely to include children suffering from internalizing and externalizing concerns compared to families categorized as experiencing “security and optimal relationships” [48]. Notably, although emotional security theory stresses the role of emotional security as a mechanism accounting for the impact of sociopolitical violence exposure on families, scant research has examined the impact of sociopolitical violence on *parental* emotional security in the family. Moreover, even less is known about the distinctive effects of sociopolitical violence on maternal and paternal emotional security, specifically, and the specific implications of each parents’ emotional security on adolescent adjustment.

Exploring the relationship between parental emotional security within the family setting is an important step in further understanding elements of the family system that may contribute to youth mental health and psychosocial concerns. Understanding the family system and the inherent risk and protective factors present within the system, is especially important in areas affected by chronic conflict. Aitcheson et al. (2017) found that among a sample of Gazan students, those who reported a stronger family coherence experienced fewer symptoms of depression and anxiety [49]. Emotional security theory holds that contexts of sociopolitical violence are likely to have implications for the emotional security of all family members, and that the emotional security of each of the parents in families are expected to be related to adolescent adjustment [50]. The present study breaks new ground in examining these important questions related to the impact of parental emotional security and sociopolitical violence exposure on adolescent adjustment.

### 1.4. Parental Mental Health

Parental mental health has been largely established as a contributor to children’s mental health and psychosocial outcomes across diverse settings [51,52,53,54,55,56]. Studies in Gaza show that both maternal and paternal mental health difficulties, such as posttraumatic stress and anxiety, are associated with poorer mental health outcomes among their children [10,45,57]. Additionally, studies of Gazan families have found mixed results in whether good parental mental health moderates the negative impact of direct trauma on children’s mental health [45,58]. The current study further explores the relation of both maternal and paternal mental health with poor adolescent mental health, when considered alongside the potential impact of emotional security and trauma exposure within the family system.

### 1.5. The Current Study

Although a substantial amount of literature exists on the relationship between adolescents and one of their parents, few studies have incorporated both mothers and fathers in the data collection process [10,45,58]. However, understanding parents as having unique influences on their children is essential in order to effectively inform family-based mental health initiatives, especially in conflict-affected settings. Thus, the current study seeks to further explore the intergenerational implications of community violence exposure on the family system and young people’s development. This study will examine the associations between mothers’ and fathers’ traumatic experiences, maternal and paternal mental health, and maternal and paternal emotional security with parent-reported adolescent adjustment. Since the focus of the current study is on the *intergenerational* factors associated with youth adjustment, the adolescent self-report variables of trauma exposure and security in the family will serve primarily as control variables. This will promote understanding of how the experiences of mothers and fathers may independently contribute to their children’s adjustment outcomes.

Our specific hypotheses are as follows: (1) Fathers will report significantly greater violence exposure than mothers. While there is some literature that supports gender differences in trauma exposure among Palestinian adults [59,60], it is important to continue to evaluate whether this pattern hold across time. We predict this pattern will hold among adults in the current study. (2) Consistent with previous literature on the impact of parental mental health on Gazan youth [10,57], elevated rates of both maternal and paternal depression will be positively associated with greater adolescent psychological adjustment difficulties. (3) Due to the great influence of emotional security within the family system, both maternal and paternal emotional insecurity within the family will be associated with greater adolescent adjustment difficulties. (4) While previous literature presents mixed findings on the impact of maternal and paternal trauma exposure on youth outcomes in Gaza [45,46], since fathers experience elevated rates of trauma exposure [59,60], only paternal trauma exposure is expected to be associated with adolescent adjustment difficulties.

## 2. Materials and Methods

### 2.1. Participants

The current study included *N* = 68 family units (mother, father, adolescent). Therefore, 204 total individuals participated in the current study. Participating adolescents ranged in age from 13–16 years and had a mean age of 14.03 years (*SD* = 1.09). Additionally, 57.4% of adolescents identified as male (see Table 1). Fathers ranged in age from 35–65 years, with an average age of 47.03 years (*SD* = 6.84), and 27.9% of fathers reported having a high school degree or schooling beyond high school. Mothers ranged in age from 30–59 years and reported a mean age of 41.14 years (*SD* = 6.61), with 27.9% of mothers also reporting having a high school degree or schooling beyond high school. Mothers reported an average income of 386.43 New Israeli Shekels (*SD* = 324.79) in the previous month (equivalent to 116.03 USD), while fathers reported a significantly higher income of 849.03 New Israeli Shekels (*SD* = 565.90) in the previous month (equivalent to 254.94 USD; *t* (77.62) = 4.64, *p* < 0.001).

### 2.2. Procedures

The current study analyzes baseline data collected from a pilot randomized controlled trial evaluating the *Promoting Positive Family Futures* (PPFF) Program in Gaza City, Gaza. In order to participate, families had to have an adolescent child between the ages of 13 and 16 and caregiver(s) willing to participate in both the research assessments and intervention. Only families within identified catchment areas of the implementing partner, Catholic Relief Services (CRS), were eligible to participate. Catchment areas were identified in partnership with community leaders. If families indicated an interest in participating in the research study, a CRS staff member provided a brief description of study involvement. If the family remained interested, they were added to a study registry and thereafter scheduled for a baseline interview. All interviews were conducted in person at the families’ place of residence. In order to maximize family comfort, one male and one female interviewer attended each family interview, and interviews were conducted in a private or quiet space of the home by a gender-matched interviewer. Mothers and fathers completed a written electronic informed consent, and one parent completed a consent for adolescent participation. Adolescents completed a written electronic informed assent. All participants were informed that they could skip any items they wished and could withdraw from the study at any time, with no consequences for their ability to seek other services through CRS.

All baseline interviews were completed during November–December of 2019. During baseline data collection, from 12–14 November 2019, clashes between Israel and Gaza escalated. The Israeli Defense Forces orchestrated air strikes against Gaza City, which led to an estimated 34 casualties, along with 111 wounded [61]. Although a majority of participants (*n* = 53 family units) completed the baseline surveys post-escalation of this violence, some participants (*n* = 15 family units) completed the baseline survey pre-escalation.

### 2.3. Measures

All measures were administered to participants in Arabic. Instruments not already available in Arabic were translated from English to Arabic by bilingual members of the research team and then back translated to ensure accurate representation of measures and constructs. Discrepancies between back translated and original versions of the measures were highlighted and then were discussed by the full research team to determine what phrasing best retained semantic equivalence.

#### 2.3.1. Parent and Adolescent Trauma Exposure

A nine-item study-specific survey was developed to measure trauma exposure and was based on the previously developed measures *Life Events Checklist* (LEC-5) and *Gaza Traumatic Events Checklist* [62,63]. The current measure went through several rounds of revision with community partners in order to ensure cultural sensitivity and protect the safety of study participants. The finalized instrument asked participating mothers, fathers, and adolescents to indicate if they had *witnessed* or *experienced* each event, including: (1) Transportation accident (for example, car accident, boat accident, train wreck, plane crash); (2) Sexual assault, use the description: being threatened or harmed in an unethical way or being asked to do something physical that is ethically inappropriate; (3) Exposed to tear gas; (4) Life threatening illness or injury; (5) House sealed or demolished; (6) Beaten by Israeli army or settlers; (7) Stripped in public, use the description: forced to take off your clothes in a public place (the Israeli soldiers sometimes ask people to take off their clothes when evacuating their homes); (8) Had to leave my home due to conflict; (9) Hosted a displaced family due to conflict. For each participant, endorsements were tallied to create a sum score for total witnessed events (possible range 0–9), total experienced events (possible range 0–9) and total overall trauma exposure (possible range 0–18).

#### 2.3.2. Parent and Adolescent Security in the Family

An Arabic-translated version of the Security in the Family Scale (SIFS) was administered to fathers, mothers, and adolescents [64]. Participants were asked to indicate agreement with 24 items on a Likert scale from 1 (*strongly disagree*) to 5 (*strongly agree*). The measure evaluated family processes and how individuals viewed the stability and support of their families. 16 items on the assessment were reverse scored, and then a total sum score was calculated, with higher scores indicating greater feelings of security. Previous literature has shown this measure to be reliable across diverse settings, including Northern Ireland, Iran, and Spain [65,66,67]. Internal reliability was calculated in the current study using Cronbach’s Alpha (Mothers, α = 0.84; Fathers, α = 0.75; Adolescents, α = 0.81).

#### 2.3.3. Parental Depression

The Arabic version of the Patient Health Questionnaire (PHQ-9) was administered to mothers and fathers to evaluate the prevalence of parental depressive symptoms [68]. The measure has been previously validated in Arabic [44,69,70]. This measure includes nine items that describe symptoms of depression, including mood and sleep disruptions, changes in energy levels and eating habits, and thoughts of death. The final item from the PHQ-9, which asks about thoughts of death or self-harm was omitted in the current study due to concerns about sensitivity. Participants were asked to indicate how frequently they experienced each symptom in the previous two weeks by choosing from the following responses: 0 (*not at all*), 1 (*several days*), 2 (*more than half the days*) and 3 (*nearly every day*). The measure was scored by summing participant responses for a total score, with higher scores reflecting more depressive symptoms. Internal reliability was calculated using Cronbach’s Alpha (Mothers, α = 0.65; Fathers, α = 0.78).

#### 2.3.4. Parent-Reported Adolescent Adjustment

Adolescent psychological adjustment was evaluated through the Arabic-translated version of the parent-report Strengths & Difficulties Questionnaire (SDQ) [71]. This measure has previously been validated in Arabic by Alyahri and Goodman (2006) [72]. Both mothers and fathers completed the instrument, which listed 25 items related to their children’s emotional and behavioral experiences in five domains: Emotional problems, Conduct problems, Hyperactivity, Peer problems and Prosocial behavior. Five items were reverse scored, and then a total sum score for adjustment problems was calculated by summing the four core symptom subscales. Higher scores greater total adjustment difficulties. Internal reliability was calculated using Cronbach’s alpha (Mother report, α = 0.76; Father report, α = 0.71).

### 2.4. Analytic Plan

Hypothesis 1 was evaluated using two-sample t-tests, in order to understand gender differences among parental reports of violence exposure, security in the family, and depression. Hypotheses 2, 3, and 4 were evaluated simultaneously using linear regression models to evaluate how maternal and paternal trauma exposure, depression, and security in the family contribute to adolescent psychological adjustment. Since mothers and fathers independently reported on adolescent adjustment, two linear models were conducted, one model for each reporter. The first model evaluated how maternal trauma exposure, maternal depression, and maternal security in the family contributed to maternal-reported adolescent adjustment, while controlling for adolescent total overall trauma exposure, adolescent security in the family, and whether the maternal report was collected pre- or post-violence escalation. The second model evaluated how paternal trauma exposure, paternal depression, and paternal security in the family contributed to paternal-reported adolescent adjustment, while controlling for adolescent total overall trauma exposure, adolescent security in the family, and whether the paternal report was collected pre- or post-violence escalation. Given the focus of the current manuscript on the *intergenerational* implications of community violence exposure on the family system, the administered adolescent self-report measures, which measure adolescent-reported community trauma exposure and security in the family, were used primarily as control variables. By controlling for adolescents’ trauma exposure and feelings of security in the family, the current study seeks to evaluate the independent influence of parents’ experiences on their children.

Following the testing of the primary hypotheses, post hoc mediation models were developed for both the maternal and paternal models. Both the maternal and paternal mediation models evaluated whether parental depression and parental security in the family mediated the relationship between parental trauma exposure and parent-reported adolescent adjustment, while controlling for adolescent self-reported trauma exposure, adolescent self-reported security in the family, and whether the parent completed the measure pre- or post-escalation of violence.

All descriptive and multiple regression analyses were conducted using SPSS (version 27.0, IBM Corp, Armonk, NY, USA). Post hoc mediation models were conducted using STATA (version 16.0, StataCorp, College Station, TX, USA). 

## 3. Results

### 3.1. Adolescent Outcomes

On average, adolescents self-reported reported witnessing between 2 and 3 traumatic events in their lifetime (*M* = 2.98, *SD* = 1.80) and directly experiencing between 1 and 2 traumatic events (*M* = 1.46, *SD* = 1.47; see Table 2). Only 2 adolescents reported no trauma exposure, having neither witnessed nor directly experienced any of the events in the administered checklist. No differences in adolescent self-reported trauma exposure were found according to sex of the adolescent, nor between adolescents who completed the measure pre-escalation of violence and those who completed the measure post-escalation. Scores of adolescent self-reported security in the family (*M* = 87.09; *SD* = 12.59) also did not differ by sex of the adolescent.

Scores of parent-reported adolescent psychological adjustment did not significantly differ between mother reported scores (*M* = 18.18, *SD* = 6.00) and father reported scores (*M* = 16.31, *SD* = 5.28). No differences in parent-reported adolescent adjustment were found between parents who completed the measure pre-escalation of violence and parents who completed the measure post-escalation. Additionally, no differences in parent-reported adolescent adjustment were found based on the sex of the adolescent.

### 3.2. Parent Outcomes

Mothers reported witnessing an average of between 4 and 5 of the inquired about traumatic events in their lifetime (*M* = 4.66, *SD* = 1.73) and directly experiencing between 2 and 3 of the inquired about events (*M* = 2.75, *SD* = 1.52). Fathers reported witnessing between 5 and 6 of the inquired about events (*M* = 5.07, *SD* = 2.57) and directly experiencing between 3 and 4 events (*M* = 3.91, *SD* = 1.86). All parents reported at least one form of trauma exposure, either witnessed or directly experienced. In support of Hypothesis 1, fathers reported significantly greater total overall trauma exposure compared to mothers (*t* (115.91) = 2.71, *p* = 0.008). However, while fathers reported directly experiencing more traumatic events than mothers (*t* (134) = 3.99, *p* < 0.001), there was no difference between mothers and fathers in the number of events they reported witnessing.

When the community trauma exposure measure was explored at the level of individual items, it was found that fathers endorsed having directly experienced being “beaten by Israeli army or settlers” significantly more than mothers (*X*^2^(1, 136) = 48.10, *p* < 0.001), having directly experienced a “life threatening illness or injury” significantly more than mothers (*X*^2^(1, 136) = 9.67, *p* = 0.002), and having directly experienced being “stripped in public” significantly more than mothers (*X*^2^(1, 136) = 19.78, *p* < 0.001). Fathers also endorsed having witnessed a “transportation accident” (*X*^2^(1, 136) = 19.32, *p* < 0.001) and someone else be “stripped in public” (*X*^2^(1, 136) = 14.34, *p* < 0.001) significantly more than mothers, while mothers endorsed having witnessed someone “leave [their] home due to conflict” (*X*^2^(1, 136) = 4.50, *p* = 0.034) and someone “[host] a displaced family due to conflict” (*X*^2^(1, 136) = 6.20, *p* = 0.013) significantly more than fathers.

Mothers who completed the measure post-escalation of community violence indicated no differences compared to mothers who completed the measure pre-escalation. However, fathers who completed the measure post-escalation indicated witnessing significantly more traumatic events (*M* = 5.60, *SD* = 2.25) than fathers who completed the measure pre-escalation (*M* = 3.20, *SD* = 2.83; *t* (66) = 3.45, *p* < 0.001). There was no reported difference in the number of traumatic events directly experienced between fathers who completed the measure post-escalation compared to pre-escalation. Overall trauma exposure reported by fathers, however, was significantly greater post-escalation (*M* = 9.60, *SD* = 3.64) compared to pre-escalation (*M* = 6.80, *SD* = 4.55; *t* (66) = 2.49, *p* = 0.015).

Mothers (*M* = 11.76, *SD* = 4.62) and fathers (*M* = 11.69, *SD* = 5.17) reported no difference in average levels of depression. Fathers (*M* = 88.22, *SD* = 9.73) reported more security in the family compared to mothers (*M* = 79.68, *SD* = 13.70), with higher scores indicating greater feelings of security (*t* (120.89) = 4.19, *p* < 0.001). No differences in security in the family or depression were found between parents who completed the measure pre-escalation of violence and those who completed the measure post-escalation.

### 3.3. Factors Associated with Adolescent Psychological Adjustment

The regression model examining maternal report of adolescent adjustment was significant overall and explained a large amount of the variance (*F*(6) = 7.44, *R*^2^ = 42.70%, *p* < 0.001). In alignment with Hypotheses 2 and 3, maternal depression (*β* = 0.31, *p* = 0.011) and maternal security in the family (*β* = −0.36, *p* = 0.004) were shown to significantly contribute to mother reported adolescent psychological adjustment, with higher rates of maternal depression associated with higher rates of adolescent adjustment difficulties and higher rates of maternal security in the family associated with lower rates of adolescent adjustment difficulties (see Table 3). With regard to Hypothesis 4, maternal trauma exposure did not significantly contribute (*β* = −0.07, *p* = 0.517) to adolescent psychological adjustment. Thus, Hypotheses 2 and 3 were supported within the maternal regression model.

The regression model examining paternal report of adolescent adjustment was also significant overall (*F*(6) = 4.57, *R*^2^ = 31.00%, *p* < 0.001). Here, however, only paternal trauma exposure significantly contributed to father reported adolescent psychological adjustment (*β* = 0.32, *p* = 0.012), with higher rates of paternal trauma exposure being associated with higher rates of adolescent adjustment difficulties (see Table 4). Paternal depression (*β* = 0.15, *p* = 0.268) and paternal security in the family (*β* = −0.27, *p* = 0.057) did not significantly contribute to adolescent psychological adjustment. Thus, for fathers, Hypotheses 2 and 3 were not supported since paternal depression and security in the family were not significant, while Hypothesis 4 was supported since paternal trauma exposure was significantly associated with adolescent adjustment difficulties.

### 3.4. Post hoc Analyses

**Figure 1:**
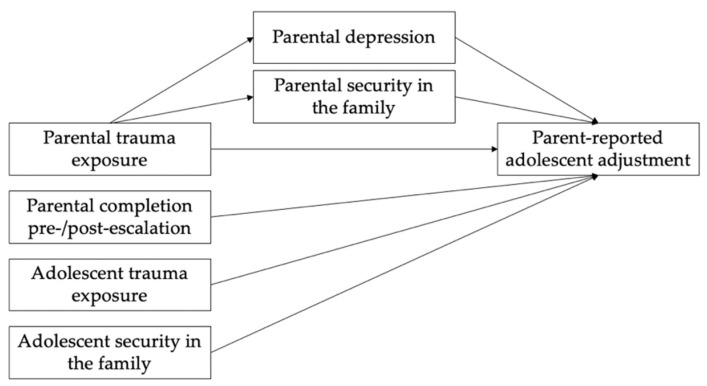
Tested mediation models. A model with maternal variables and a model with paternal variables were tested separately.

Post hoc cross-sectional mediation models were also examined. The first cross-sectional mediation model evaluated whether paternal depression and paternal security in the family mediate the relationship between paternal trauma exposure and father reported adolescent adjustment, while controlling for adolescent self-reported trauma exposure, adolescent self-reported security in the family, and whether the father completed the measure pre- or post-escalation of violence (see Figure 1). The model showed a significant direct effect of paternal trauma exposure on father reported adolescent adjustment (*β* = 0.42, *p* = 0.006, 95% CI [0.129, 0.721]). There was also a significant indirect effect of paternal trauma exposure on father reported adolescent adjustment through the mediator of paternal security in the family, with a significant relationship between paternal trauma exposure and paternal security in the family (*β* = −0.88, *p* = 0.001, 95% CI [−1.423, −0.344]), as well as between paternal security in the family and father reported adolescent adjustment (*β* = −0.14, *p* = 0.040, 95% CI [−0.281, −0.006]). Additionally, the model showed a significant relation between paternal trauma exposure and paternal depression (*β* = 0.31, *p* = 0.044, 95% CI [0.008, 0.605]), but not between paternal depression and father reported adolescent adjustment. Finally, the model also found a direct relation between whether the father completed measures pre- or post-escalation of violence and father reported adolescent adjustment (*β* = −2.73, *p* = 0.043, 95% CI [−5.375, −0.089]).

The second mediation model evaluated whether maternal depression and maternal security in the family mediate the relationship between maternal trauma exposure and maternal reported adolescent adjustment, while controlling for adolescent self-reported trauma exposure, adolescent self-reported security in the family, and whether the mother completed the measure pre- or post-escalation of violence. This model confirmed that maternal trauma exposure was not directly associated with mother reported adolescent adjustment, nor was it significantly associated with either maternal depression or maternal emotional security in the family. However, maternal security in the family (*β* = −0.16, *p* = 0.002, 95% CI [−0.252, −0.058]) and maternal depression (*β* = 0.40, *p* = 0.006, 95% CI [0.118, 0.685]) were both significantly associated with mother reported adolescent adjustment.

## 4. Discussion

The current study sought to evaluate how intergenerational factors may influence Gazan adolescents’ adjustment outcomes. More specifically, the study evaluated how Gazan mothers’ and fathers’ trauma exposure, emotional security, and mental health were associated with their adolescents’ psychological adjustment. Adolescents and their parents all reported high rates of violence exposure. Of the nine items included in the study-specific survey on trauma exposure, adolescents reported witnessing an average of 2–3 of the traumatic events in their lifetime and directly experiencing 1–2 events, while mothers reported witnessing an average of 4–5 traumatic events and experiencing 2–3 events, and fathers reported witnessing an average of 5–6 traumatic events and experiencing 3–4 events. As anticipated in Hypothesis 1, and consistent with previous literature [59,60], fathers reported higher total overall trauma exposure compared to mothers. However, although fathers reported directly *experiencing* more traumatic events than mothers, there was no difference between mothers and fathers in the number traumatic events they reported *witnessing*. This finding may be due to males facing more frequent direct contact with Israeli soldiers, as evident through examination of individual items on community trauma exposure. Specifically, results indicated that fathers reported being beaten by Israeli soldiers more than mothers and being stripped in public more than mothers. In studies of Palestinian youth, young boys have also reported experiencing significantly greater violence exposure than girls [41,73,74].

Mothers and fathers also reported no difference in depressive symptoms, which is consistent with previous findings among Palestinian adults across Gaza, the West Bank, and East Jerusalem [42,75]. However, fathers reported greater feelings of security in the family than mothers. Previous literature on security in the family has also primarily been focused on children rather than parents, so this finding is a particularly interesting contribution.

Hypotheses 2 through 4 postulated that parental depression and security in the family would significantly contribute to parent-reported adolescent psychological adjustment, while parental trauma exposure would contribute to adolescent adjustment within only the paternal model. However, the findings only supported Hypotheses and 3 for the maternal model. The regression model examining maternal reports did find that as maternal depression increased, mother reported adolescent adjustment difficulties also increased, and as maternal feelings of security in the family increased, mother reported adolescent adjustment difficulties decreased. There was also no relationship found between maternal trauma exposure and mother reported adolescent adjustment. This finding was further supported through the post hoc maternal mediation model, which showed that maternal trauma was not directly associated with mother reported adolescent adjustment, nor was it associated with maternal depression or maternal security in the family. Maternal security in the family and maternal depression, however, were both directly associated with mother reported adolescent adjustment. This is a particularly interesting finding, and further research should continue to explore the relationships between maternal internalizing symptoms, violence exposure, and youth outcomes. Therefore, Hypotheses 2 and 3 were supported within the maternal regression model.

However, in contrast with the results for Hypotheses 2 and 3 in the maternal model, tests of the paternal model found no relationship between paternal depression and father reported adolescent adjustment nor between paternal security in the family and father reported adolescent adjustment. The paternal model did support Hypothesis 4, and showed that showed that as paternal trauma exposure increased, father reported adolescent adjustment difficulties also increased. The post hoc paternal mediation model supported the direct relationship between paternal trauma exposure and father reported adolescent adjustment, and also showed that paternal trauma exposure may indirectly influencing adolescent adjustment through paternal security in the family. This finding shows that while the effects of trauma exposure may operate through differing processes among mothers and fathers, supporting both paternal and maternal feelings of security in the family may be especially important in promoting adolescent psychological adjustment outcomes.

This study is a particularly important contribution in understanding how family system variables inform adolescent well-being within the context of Gaza, particularly in its incorporation of the traumatic experiences and mental health of multiple family members. These findings support social–ecological theory’s assertions of the importance of exploring the impact of multiple social levels on child outcomes [76], and further demonstrate that emotional security and the intergenerational transmission of trauma are important frameworks for better understanding adolescent development and psychosocial wellbeing. However, despite the large influence of parents on their children’s mental health, few studies in Gaza incorporate both the mother and father into the data collection process. This study displays how both mothers and fathers influence adolescent adjustment, which is essential to understand when informing the development of family-based initiatives that involve both parents. If the unique contributions of Gazan mothers and fathers to adolescent wellbeing are better understood, the content of family-based mental health initiatives developed within the Palestinian context may be more closely tailored towards the unique experiences of different parents.

The study provides evidence that research focused on the unique contributions of each parent towards their children’s socioemotional development should continue to be pursued throughout conflict-affected areas. Although the focus of the current study is in Gaza, Palestine, this study has implications for mental health research globally. Culturally tailored family-based mental health initiatives have been shown across literature to be promising in promoting the psychosocial outcomes of young people, even in settings affected by sociopolitical violence [77,78]. However, if mothers and fathers are differentially influencing their children’s adjustment outcomes, it may be additionally beneficial for intervention content to be tailored specifically towards the unique needs of mothers and fathers, rather than assuming homogeneity in needs across all parents.

It is also essential to consider what can be addressed at the level of the mental health practitioner through program development. Parental concerns such as depression and security in the family may be feasible to address in family-based mental health interventions, but the impact of paternal violence exposure on adolescent adjustment highlights the need for the mental health field to recognize and address the ongoing impact of direct and structural forms of violence on the wellbeing of young people, and recognize that violence prevention is critical to the health and wellbeing of families [79].

### Limitations and Future Directions

The current study focused on the experiences of families living in Gaza, many children around the world live in conflict-affected settings [1]. Future research focused on families and intergenerational processes may shed valuable light on variation and consistency in these associations across contexts. The current study used baseline data from a pilot of a randomized controlled trial. Therefore, the small sample size of *N* = 68 family units serves as a limitation and future studies should continue to explore the unique contributions of mothers and fathers to adolescent adjustment. Additionally, the current study measured the construct of adolescent *psychological adjustment* solely through a parent-report measure. It would be helpful for future studies to also administer self-report scales on adolescent psychological adjustment, to minimize potential bias. As a study based on baseline data for a pilot of a randomized trial, the evaluation of relations is necessarily cross-sectional, which limits the interpretation of causality. Future research should examine these constructs over time, which will allow for more robust analyses, including the examination of intraindividual trajectories over time. Future research should also consider how the differential impact of parental variables on youth mental health can be used to inform parent-specific intervention efforts, and how treatment initiatives may provide unique content to mothers and fathers in order to best support the needs of their children and families.

## 5. Conclusions

The current study explored how parental trauma exposure, parental emotional security, and parental mental health contribute to adolescent psychological adjustment among families living in the Gaza Strip. Maternal depression and security in the family were significantly associated with their adolescents’ psychological adjustment, while paternal trauma exposure was significantly associated with their adolescents’ psychological adjustment. These findings suggest that mothers and fathers may differentially inform their children’s socioemotional development. By further understanding family system functioning within areas affected by chronic conflict and structural violence, mental health professionals can provide better care to young people and their families in order to promote more positive socioemotional outcomes.

## Figures and Tables

**Table 1:** Demographic variables of study participants.

Demographic Variables	Mothers (n = 68)	Fathers (n = 68)	Adolescents (n = 68)
Age	*M* = 41.1 years	*M* = 47.0 years	*M* = 14.0 years
Sex	*-*	*-*	57.4% male
Education			
*Grade school or less*	48.5%	39.7%	-
*Some high school*	23.5%	32.4%	-
*High school degree/GED*	13.2%	13.2%	-
*Education beyond high school*	14.7%	14.7%	-
Currently working?	Yes (4.4%)	Yes (45.6%)	-

**Table 2:** Correlation Matrix.

Variable	1	2	3	4	5	6	7	8	9	10	11	12
Maternal trauma exposure	1.00											
2. Maternal depression	0.09	1.00										
3. Maternal SIF	0.00	−0.52 **	1.00									
4. Maternal participation pre-/post-escalation	0.18	0.15	−0.00	1.00								
5. Paternal trauma exposure	0.11	0.16	−0.30 *	0.29 *	1.00							
6. Paternal depression	0.23	0.19	−0.06	0.06	0.24	1.00						
7. Paternal SIF	−0.28 *	−0.19	0.28 *	−0.13	−0.36 **	−0.57 **	1.00					
8. Paternal participation pre-/post-escalation	0.18	0.15	−0.00	1.00	0.29 *	0.06	−0.13	1.00				
9. Adolescent trauma exposure	−0.10	0.01	−0.12	−0.12	0.05	−0.18	−0.04	−0.12	1.00			
10. Adolescent SIF	−0.00	−0.17	0.28 *	−0.00	−0.24 *	0.03	0.05	−0.00	−0.36 **	1.00		
11. Maternal-report adolescent adjustment	−0.04	0.52 **	−0.57 **	−0.00	0.19	0.02	−0.13	−0.00	0.10	−0.33 **	1.00	
12. Paternal-report adolescent adjustment	0.11	0.31 *	−0.43 **	−0.09	0.37 **	0.35 **	−0.44 **	−0.09	0.08	−0.04	0.38 **	1.00

Note. SIF=security in the family. *. Correlation is significant at the level of *p* = 0.05 (2-tailed). **. Correlation is significant at the level of *p* = 0.01 (2-tailed).

**Table 3:** Maternal Regression Model.

	Adolescent Psychological Adjustment		
Variable	*β*	*t*	*p*-Value
Maternal trauma exposure	−0.07	−0.65	0.517
Maternal depression	0.31 *	2.63	0.011
Maternal security in the family	−0.36 **	−2.97	0.004
Maternal completion of forms pre-/post-escalation	−0.04	−0.42	0.677
Adolescent trauma exposure	−0.03	−0.25	0.802
Adolescent security in the family	−0.19	−1.71	0.093

Note. *F* = 7.44, *R*^2^ = 42.70%, *p* < 0.001. * Significant at the level of *p* = 0.05 (2-tailed). ** Significant at the level of *p* = 0.01 (2-tailed).

**Table 4:** Paternal Regression Model.

	Adolescent Psychological Adjustment		
Variable	*β*	*t*	*p*-Value
Paternal trauma exposure	0.32 *	2.59	0.012
Paternal depression	0.15	1.12	0.268
Paternal security in the family	−0.27	−1.94	0.057
Paternal completion of forms pre-/post-escalation	−0.22	−1.92	0.060
Adolescent trauma exposure	0.08	0.66	0.511
Adolescent security in the family	0.07	0.62	0.538

Note. *F* = 4.57, *R*^2^ = 31.00%, *p* < 0.001. * Significant at the level of *p* = 0.05 (2-tailed).

## Data Availability

The data are not publicly available as participants did not consent to this. Data are available on request and with the approval of participating agencies, with appropriate measures taken to protect the privacy of participants. Please contact the corresponding author with any requests for information.
